# Tulane virus protease as a structural surrogate for inhibitor screening of human norovirus proteases

**DOI:** 10.1128/jvi.02176-25

**Published:** 2026-02-11

**Authors:** Son Pham, Nikhil Sharma, Banumathi Sankaran, Jalen Nguyen, Mary K. Estes, Joseph M. Hyser, B. V. Venkataram Prasad

**Affiliations:** 1 Verna and Marrs McLean Department of Biochemistry and Molecular Pharmacology, Baylor College of Medicine 3989, Houston, Texas, USA; 2 Department of Molecular Virology and Microbiology, Baylor College of Medicine 3989, Houston, Texas, USA; 3 Berkeley Center for Structural Biology, Molecular Biophysics, and Integrated Bioimaging, Lawrence Berkeley Laboratory 1666 , Berkeley, California, USA; 4 Department of Medicine, Baylor College of Medicine 3989, Houston, Texas, USA; 5 Alkek Center for Metagenomics & Microbiome Research, Baylor College of Medicine 3989, Houston, Texas, USA; Dartmouth College Geisel School of Medicine, Hanover, New Hampshire, USA

**Keywords:** noroviruses, proteases, protease inhibitors, crystal structures, inhibitor design, inhibition assays

## Abstract

Human norovirus (HuNoV) is a significant cause of gastroenteritis worldwide, affecting people of all age groups. There are currently no vaccines or drugs available, leaving susceptible populations vulnerable to severe or protracted illness. A HuNoV cultivation system is pivotal for screening norovirus antivirals. While the human intestinal enteroid cultivation system allows robust replication of multiple HuNoV strains, it presents technical and cost barriers. Tulane virus (TV), a surrogate for HuNoV, replicates well in monkey kidney cell lines and is closely related to norovirus in cellular biology. Here, we determined the structures of TV protease (TV-Pro) alone and in complex with rupintrivir, a picornavirus inhibitor that also inhibits HuNoV proteases (HuNoV-Pro). Our data validate TV as an efficient surrogate system for rapid screening of HuNoV protease inhibitors. The TV protease structure exhibits significant backbone similarity to the GI.1 HuNoV protease in the substrate-binding domain, with the BII-CII loop in an open conformation stabilized by hydrogen bonds as present in the GI.1 protease. Structural differences in the S2 pocket and two amino acid changes in the S4 pocket result in slightly altered P2 and P4 substrate and inhibitor conformations. Despite these differences, we confirm previous findings that the TV protease can cleave the GI.1 and GII HuNoV polyprotein substrates with high and moderate efficiency, respectively. We found that rupintrivir efficiently inhibits TV protease *in vitro* and inhibits TV replication in cell culture with similar efficacy in combination with P-glycoprotein efflux pump inhibitors. We conclude that TV is a valuable surrogate for HuNoV protease inhibitor screening and outline strategies to improve its compatibility as such.

**IMPORTANCE:**

Human noroviruses (HuNoVs) are a significant cause of sporadic and epidemic gastroenteritis worldwide. There are no vaccines or antiviral drugs currently available to treat infections. Our work here demonstrates the potential of the Tulane virus cell culture system as a surrogate for screening small-molecule inhibitors of the human norovirus proteases.

## INTRODUCTION

Human norovirus (HuNoV), a member of the Caliciviridae family, is a major causative agent of sporadic and epidemic viral gastroenteritis worldwide (1, 2). The burden imposed by HuNoV includes almost ~700 million infections and ~200,000 deaths annually, as well as over 4 billion dollars in direct healthcare costs and 56 billion dollars in productivity losses each year (3). In older adults and immunocompromised individuals, HuNoV cases are often more severe and have a prolonged course of illness (4, 5). However, there are currently no vaccines or drugs available for norovirus. Thus, the development of targeted HuNoV antivirals is essential to treat HuNoV infections in these vulnerable populations.

A major bottleneck in the screening of HuNoV antivirals is assessing their effectiveness against cell culture infection models. Cellular screens for HuNoV protease (HuNoV-Pro) inhibitors historically used norovirus replicons (6–8), which are helpful but not representative of a bona fide infection. While infectious models for HuNoV such as murine norovirus, zebrafish larvae, human intestinal organoids (HIOs), and human intestinal enteroids (HIEs) exist, each model has its own challenges. Murine norovirus propagates poorly in human cell lines without the expression of the cognate receptor (9), whereas zebrafish larvae (10) and human intestinal organoid (11) systems have not yet produced an adequate yield of infectious particles for repeated passaging. Only recently did a breakthrough in the human intestinal enteroid system allow repeated passaging of GII.3 HuNoV and enhance the replication of several other HuNoV strains (12). The accessible, robust cultivation of the Tulane virus (TV), belonging to the Recovirus genus in the family *Caliciviridae (*1), in monkey cell lines makes it an attractive potential surrogate for drug screening in an infectious model. Analysis of the TV genome has already identified TV protease (TV-Pro) as a cysteine protease of similar size to the HuNoV protease (HuNoV-Pro) (13). Subsequent research showed that TV-Pro cleaves HuNoV polyprotein at specific cleavage sites, and conversely, HuNoV-Pro cleaves TV polyprotein (14), indicating the possibility of using the TV system as a surrogate for cellular screening of HuNoV protease inhibitors. However, before establishing it as a potential surrogate, TV-Pro must be assessed for its structural and functional compatibility with HuNoV-Pro.

In this study, we characterized the structural mechanisms of TV-Pro substrate and inhibitor binding and determined its cleavage activity against polyprotein substrates from GI and GII HuNoVs. We found TV-Pro exhibits high backbone similarity in the substrate-binding domain and can cleave both HuNoV GI and GII substrates, although there are significant shifts in the layout of the S2 and S4 pockets that lead to altered P2 and P4 substrate and inhibitor conformations. Additionally, we demonstrate that rupintrivir inhibits TV replication in cell culture. However, this inhibition had a lower efficiency than our observed *in vitro* inhibition. Yet, with the addition of P-glycoprotein inhibitors, the antiviral of rupintrivir was restored to a level similar to that of the *in vitro* inhibition efficiency.

## RESULTS

### TV-Pro exhibits high backbone similarity to HuNoV proteases in the substrate-binding domain and a putatively stable BII-CII loop like GI.1 Norwalk protease

To compare and contrast the TV-Pro with the HuNoV-Pro in terms of the structure and inhibitor binding, we determined the crystal structures of TV-Pro alone and in complex with rupintrivir at 2.1 Å and 1.9 Å resolution, respectively. The reason for choosing rupintrivir, an efficient inhibitor of picornavirus proteases (15), is that, in our previous studies, we have shown that it binds to HuNoV-Pros and inhibits their activity, with differential kinetics between GI and GII proteases involving conformational alterations (16).

**Fig 1:**
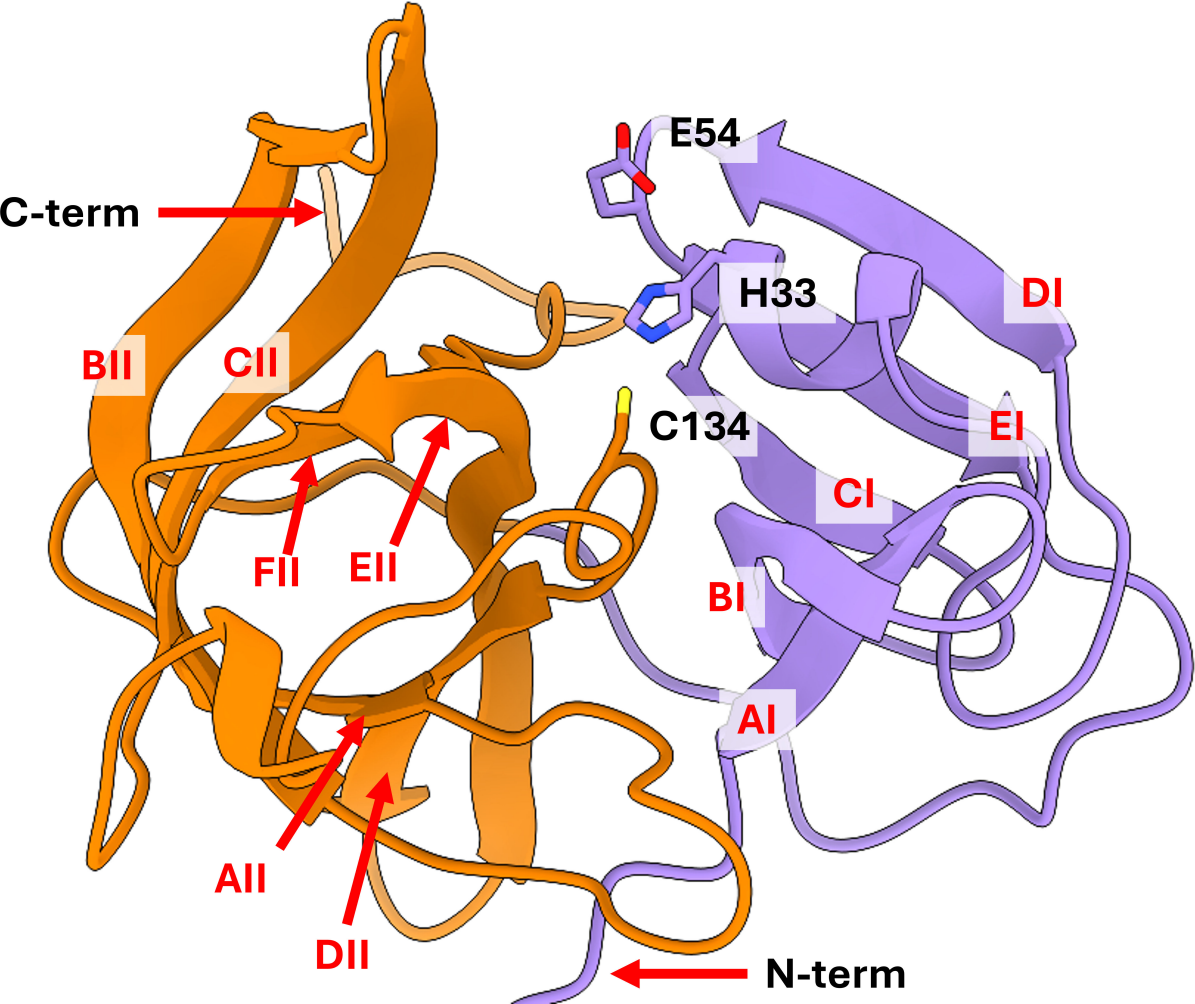
Structure and topology of Tulane virus protease. TV-Pro shares the general chymotrypsin-like protease topology with norovirus proteases and picornavirus 3C-like proteases. It consists of two beta barrel domains (first and second beta barrels in purple and orange, respectively), and the catalytic triad of cysteine, histidine, and glutamic acid (residues 134, 33, and 54, respectively) is present. Individual strands of each beta barrel are labeled in red (AI–EI and AII–FII).

The TV-Pro structure is largely similar to the structures of HuNoV-Pros, with a twin beta-barrel, chymotrypsin-like topology (Fig. 1). The first beta barrel domain is formed by five beta strands (AI–EI). A short helix, followed by a short loop, connects the CI and DI beta strands, stabilizing the twisted beta strand conformation. The same short helix positions catalytic His33 at the active site, while a short loop connecting the DI and EI beta strands orients Glu54 toward the active site. The second beta-barrel domain is formed by six beta-strands (AII–FII). The catalytic Cys134 residue is positioned within a long loop region connecting the CII and DII strands of the second beta-barrel (Fig. 1).

**Fig 2:**
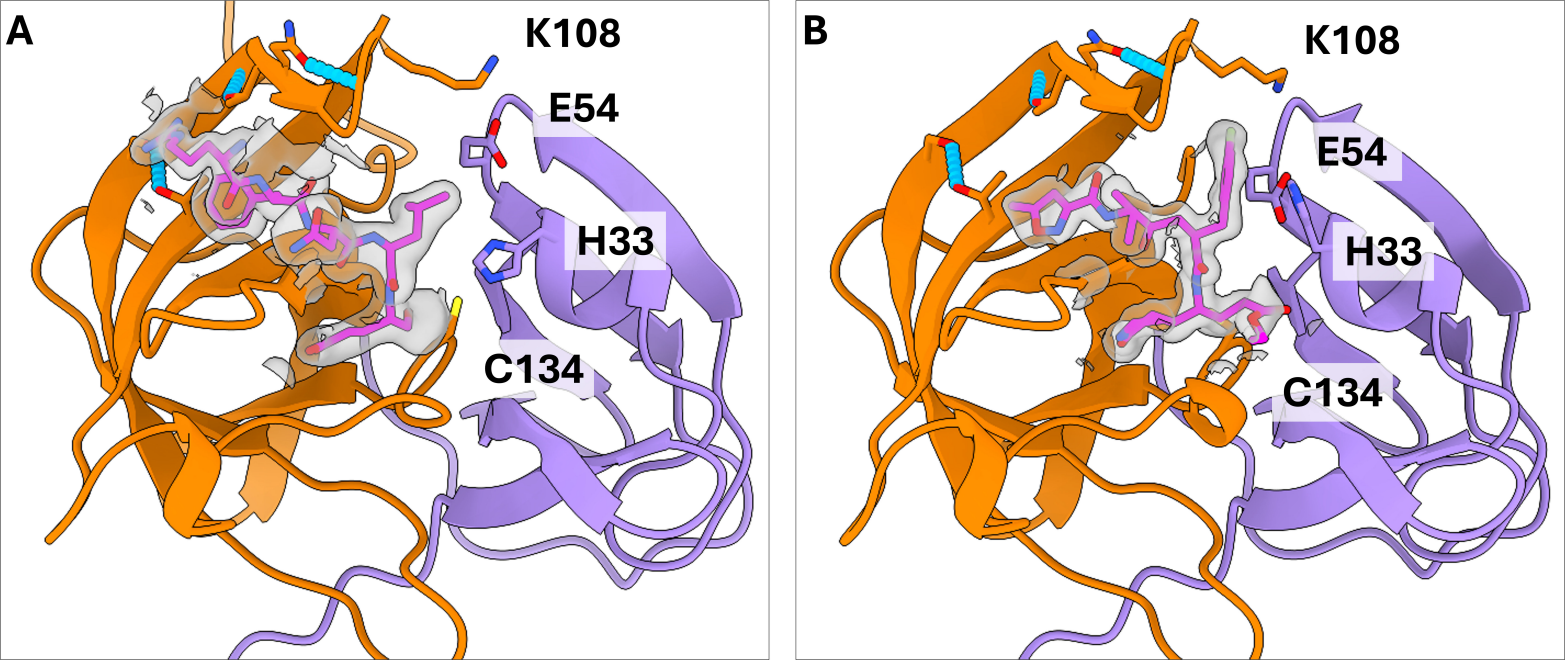
Structures of Tulane virus protease bound to its own C-terminal tail and bound to rupintrivir. (**A**) TV-Pro bound to its own C-terminal tail (magenta) originating from a neighboring subunit in the crystallographic unit cell. (**B**) TV-Pro bound to rupintrivir (magenta). In both panels, the first and second beta barrels of TV-Pro are colored in purple and orange, respectively. Hydrogen bonds stabilizing the BII-CII loop are shown in cyan. The electron densities of the ligand at the SD level of 1.0 are displayed in gray.

In the crystal structure of TV-Pro, the asymmetric unit contains two monomers. The C-terminal tail of each protease monomer is bound to the active site of the neighboring monomer in the crystal, providing the structural details of substrate binding (Fig. 2A). However, in the crystal structure of TV-Pro with rupintrivir, the asymmetric unit consists of a single subunit, with the catalytic pocket bound by rupintrivir, providing structural details of the inhibitor binding for comparison (Fig. 2B). In both structures, we observe that the BII-CII loop of the TV-Pro contains three intraloop hydrogen bonds between residues Ser99-Thr114, Gly101-Thr103, and Asn104-Gly107. No apo structures are available for TV-Pro to confirm the conformation of the BII-CII loop prior to substrate binding. However, the AlphaFold3-predicted structure of TV-Pro features all three pairs of hydrogen bonds and exhibits high similarity with our crystal structures (phase-solved using AlphaFold2 predictions). Curiously, the AlphaFold2-predicted structure shows a different rotamer for Thr103, resulting in a more open conformation for the BII-CII loop (Fig. S1). While this can be interpreted as Thr103 potentially acting as a conformational switch in the BII-CII loop, AlphaFold2 also predicts a different conformation for the peripheral loop in the S4 pocket, unlike AlphaFold3 (Fig. S1). Therefore, we postulate that the BII-CII loop in TV-Pro is rigid, like the BII-CII loop of HuNoV GI.1 protease, and in contrast to the more flexible BII-CII loop in HuNoV GII proteases.

**Fig 3:**
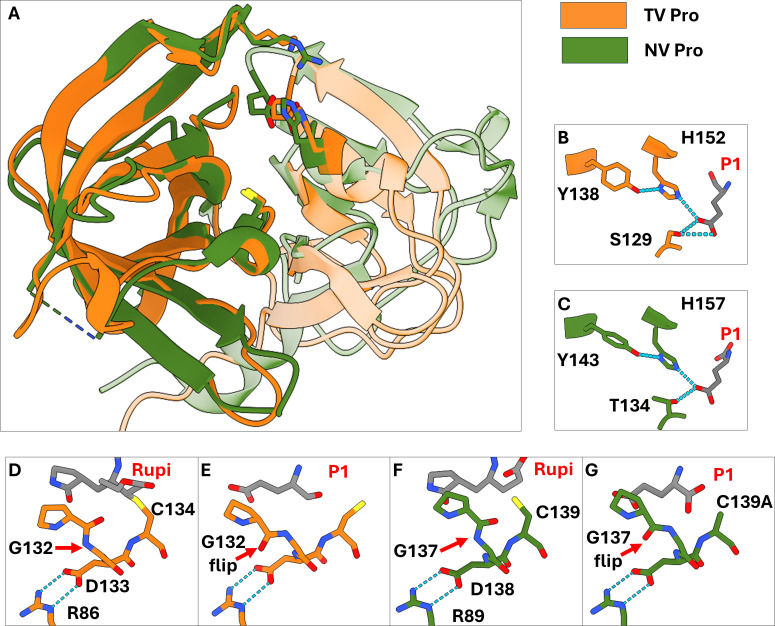
Tulane virus protease has a highly similar second beta barrel, S1 pocket, and oxyanion hole structure compared to GI.1 Norwalk human norovirus. (**A**) Overlay of TV-Pro and GI.1-Pro (PDB: 9D9Y) rupintrivir-bound structures. While the first beta barrels of TV-Pro (orange) and GI.1-Pro virus protease (green) are structurally distinct (transparent ribbon), the second beta barrels of both proteases are highly similar (colored ribbon). (**B, C**) The S1 pocket triads recognize the P1 residue in TV-Pro (orange) and GI.1-Pro (green), respectively. TV-Pro contains a Tyr-His-Ser triad, unlike GI.1-Pro, which has a threonine in the third position. (**D, F**) The backbone amides in the oxyanion hole glycine of TV-Pro (orange) and GI.1-Pro (green, PDB: 9D9Y) are not flipped when these proteases are bound to rupintrivir (gray). (**E, G**) The backbone amides in the oxyanion hole glycine of TV-Pro (orange) and GI.1-Pro (green, PDB: 4IN1) are flipped when these proteases are bound to the N-terminal substrate sequence (P1 Glu residue in gray).

While the first beta-barrel domain of TV-Pro has a markedly different structure compared to the same region in HuNoV-Pro, the second beta-barrel domain of TV-Pro is highly similar in terms of the peptide backbone structure to that of HuNoV-Pros (RMSD = 1.19Å to rupintrivir-bound HuNoV GI.1-Pro when residues 1–74 of TV-Pro are excluded, Fig. 3A). Thus, several features of the substrate-binding site of TV-Pro are virtually identical to those observed in GI.1 and GII HuNoV-Pros. The S1 pocket of TV-Pro contains a histidine-tyrosine interaction pair with identical side chain positions to those in HuNoV Pros. However, TV-Pro utilizes a His-Tyr-Ser motif (Fig. 3B) to recognize the glutamine/glutamate side chain oxygen, instead of the His-Tyr-Thr motif like HuNoV-Pro (Fig. 3C). This serine position is highly conserved in G1 recoviruses, while G3 recoviruses contain a threonine instead. The S1 pocket of TV-Pro also includes an identical Pro-Gly-Asp-Cys motif, which forms the oxyanion hole (Fig. 3D and E). Asp132 is stabilized by a salt bridge with Arg86, similar to the Asp-Arg interactions observed in HuNoV-Pro structures. In the TV-Pro structure with the bound substrate, an additional unresolved density is observed near the C-terminus, likely corresponding to the tryptophan added to the sequence to facilitate protein concentration measurements. Nonetheless, in this structure, the oxyanion hole is fully formed (Fig. 3E). Interestingly, like in the structures of GI.1-Pro and GII-Pro in complex with rupintrivir and HuNoV-Pro in complex with various peptidyl aldehyde inhibitors (17, 18), in the TV-Pro structure with rupintrivir, the backbone amides are not flipped (Fig. 3D and F). This is contrary to human rhinovirus 3C protease (15), where the oxyanion hole helps stabilize the rupintrivir warhead, suggesting that the oxyanion hole of TV-Pro behaves similarly to that of HuNoV-Pro.

### Differential orientation of S2 and S4 pockets in TV-Pro leads to altered P2 and P4 substrate and inhibitor conformations

**Fig 4:**
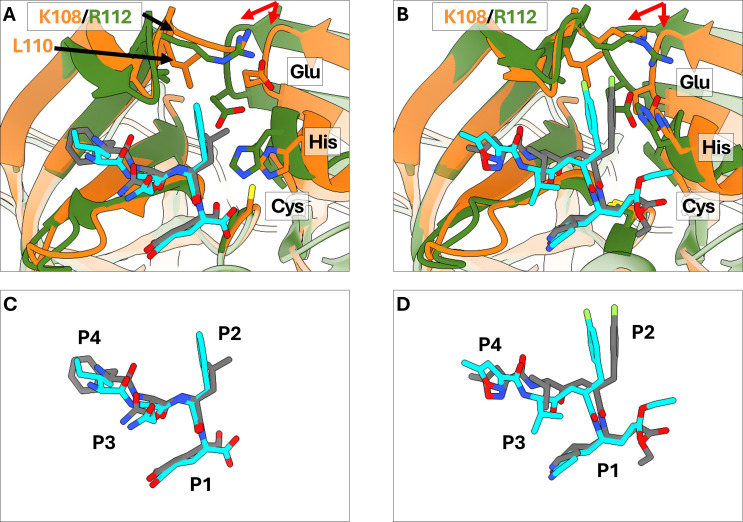
S2 pocket of Tulane virus protease is shifted compared to HuNoV protease. (**A**) Superimposed structures of TV-Pro with substrate peptide (orange, peptide in gray) and GI.1-Pro with substrate peptide (green, peptide in cyan, PDB: 4IN1). The orientation of the first beta barrel of TV-Pro is notably shifted (red arrows), subsequently resulting in the shifted orientation of the TV-Pro S2 pocket. (**B**) Superimposed structures of TV-Pro with rupintrivir (orange, rupintrivir in gray) and GI.1-Pro with sequence (green, rupintrivir in cyan, PDB: 9D9Y). The same shift in the S2 pocket here. Additionally, the P2 side chain of rupintrivir bound to TV-Pro is also shifted, presumably to conform to the altered S2 pocket of the protease. (**C**) Superimposed poses of the bound substrate peptides (TV-Pro-bound peptide in gray; GI.1-Pro-bound peptide in cyan) from panel **A**. (**D**) Superimposed poses of bound rupintrivir from panel B (from TV-Pro structure—gray; from GI.1-Pro structure—cyan), showing the shifted P2 position in TV-Pro-bound rupintrivir. As discussed in Fig. 5, both panels (**C** and **D**) show that the P4 residues in both substrate and rupintrivir bound to TV-Pro are also shifted compared to their GI.1-Pro-bound counterparts.

Due to the differential arrangement of the two beta barrels in TV-Pro compared to the HuNoV protease, the S2 pocket of TV-Pro has a noticeable tilt compared to the HuNoV protease S2 pocket (Fig. 4A and B). While the positions of the backbone amide, carbonyl, and α-carbon atoms of the P2 residue are unchanged, larger aromatic side chains are forced to adopt a shifted conformation, compared to the P2 side chain conformations in HuNoV protease structures, to match the shifted S2 pocket (Fig. 4C). This change is observed in the TV-Pro-rupintrivir structure, which shows a shifted P2 side chain conformation for rupintrivir (Fig. 4D). The change from Val114 in HuNoV protease to Leu110 in TV-Pro also contributes to the P2 conformational shift. The conserved Arg112 in HuNoV protease is replaced with Lys108 (Fig. 4A and B).

**Fig 5:**
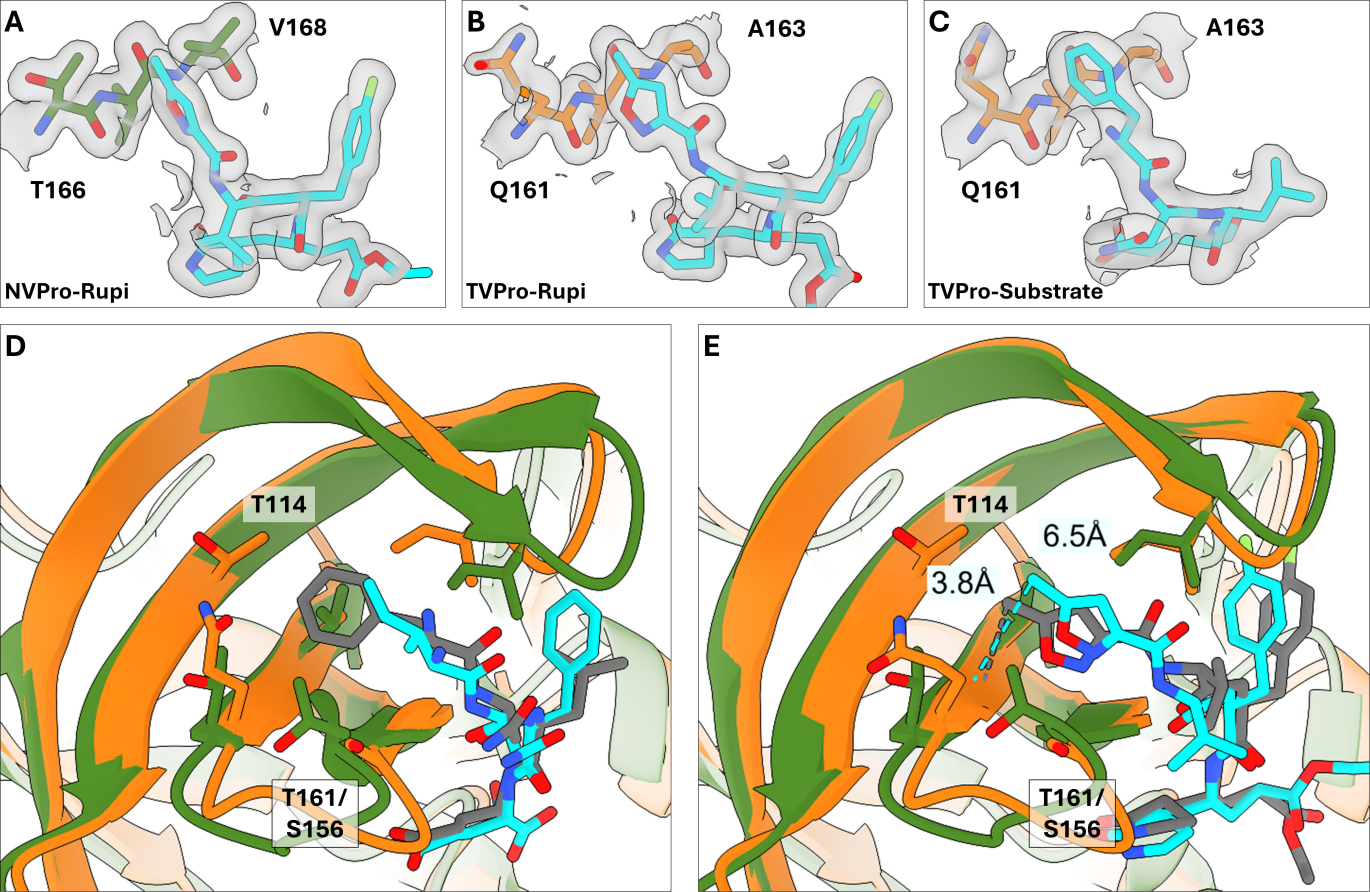
P4 residue of the substrate and rupintrivir are placed deeper into the Tulane virus protease S4 pocket compared to the HuNoV protease S4 pocket. (**A**) Electron densities at 1.0 SD level (gray) of GI.1-Pro Thr166-Ala167-Val168 (green) and bound rupintrivir (cyan). (**B,C**) Electron densities at 1.0 SD level (gray) of TV-Pro Thr161-Ala162-Ala163 (orange) and bound substrates (panel D, cyan) and bound rupintrivir (panel E, cyan). The threonine and valine may have prevented the protease from adopting the lower position in the S4 pocket, and their mutations to glutamine and alanine created a cleft that the P4 residue of substrate peptides and rupintrivir can bind. (**D**) Superimposed structures of TV-Pro with substrate peptide (orange, peptide in gray) and GI.1-Pro with substrate peptide (green, peptide in cyan, PDB: 4IN1). (**E**) Superimposed structures of TV-Pro with rupintrivir (orange, rupintrivir in gray) and GI.1-Pro with rupintrivir (green, rupintrivir in cyan, PDB: 9D9Y). The P4 residues of the TV-Pro-bound substrate (panel A) and rupintrivir (panel B) are placed deeper into the S4 pocket in TV-Pro compared to in GI.1-Pro. Distances from the methyl group carbon of rupintrivir to the alpha carbon of the underlying alanine residue in the S4 pocket confirm the differential placement of the P4 residue.

The S4 pocket of both TV-Pro and GI.1-Pro is in close proximity to the BII, CII, FII, and EII beta strands of each protease’s second beta barrel, with the FII and EII being the closest to the P4 residue. In GI.1-Pro, the S4 pocket is delineated by Thr166 and Val168 in the FII strand; these laterally facing side chains force the P4 moiety of rupintrivir into a raised conformation (Fig. 5A), and as a result, the P4 moiety of rupintrivir engages in nonbonded interactions with the outer residues of the BII-CII loop and the EII strand, Thr161, Ala160, and Ile109. In the S4 pocket of the TV protease, Thr166 and Val168 are replaced by Gln161 and Ala163, respectively (Fig. 5B and C). This amino acid change creates a novel cleft that both the P4 phenylalanine substrate side chain (Fig. 5D) and the P4 residue of rupintrivir (Fig. 5E) preferentially bind to. As a result, the P4 moiety of rupintrivir binds with a lowered conformation in the S4 pocket and interacts with Ile105 (of the BII-CII loop) and Gly155 (of the EII strand), while losing its interaction with Ser156 (equivalent to Thr161 in the EII strand of the HuNoV protease). Notably, it also forms interactions with Gln161 (of the DII strand) and Thr114 (of the CII strand). Likewise, the P4 phenylalanine side chain of the N-terminal substrate peptide also interacts with the same residues across the CII, FII, and EII strands.

The altered conformations of the P2 and P4 residues, particularly their side chains, lead to substrate and rupintrivir molecules bound to TV-Pro having a stretched conformation compared to the conformations of GI substrate and rupintrivir bound to GI.1-Pro, respectively. In addition to the S2 and S4 pocket changes, an additional amino acid difference occurs in the S3 pocket, replacing the HuNoV-conserved Gln110 with Ala107, eliminating a potential hydrogen bond often observed between Gln110 and a peptide backbone atom.

### TV-Pro cleaves both HuNoV GI and GII substrates and is inhibited by rupintrivir

**Fig 6:**
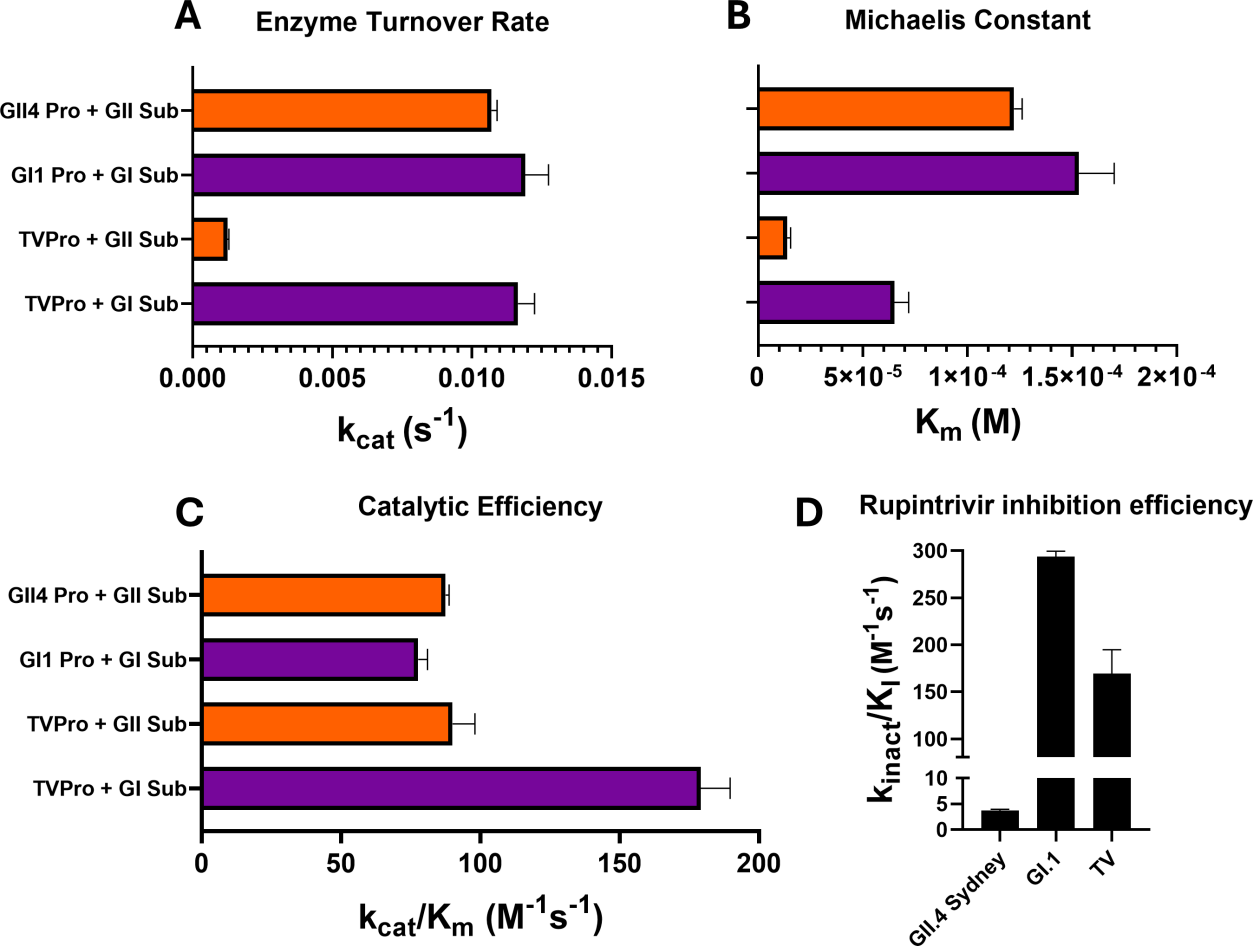
Enzymatic cleavage efficiencies of Tulane virus protease against HuNoV GI and GII substrates. (**A–C**) Enzyme kinetics constants of different pairs of proteases and substrates, from top to bottom: HuNoV GII.4 protease/HuNoV GII substrate, HuNoV GI.1 protease/HuNoV GI.1 substrate, Tulane virus protease/HuNoV GII substrate, Tulane virus protease/HuNoV GI.1 substrate. Against the HuNoV GI.1 substrate, TV-Pro displays a comparable turnover rate (kcat) and lower Michaelis constant (Km) compared to the HuNoV GI.1 protease, leading to higher catalytic efficiency (kcat/Km) overall. Against the HuNoV GII.4 substrate, TV-Pro displays much lower kcat and Km compared to the HuNoV GII.4 protease, despite showing comparable kcat/Km. (**D**) Rupintrivir covalent inhibition efficiency against HuNoV GII.4 Sydney and GI.1 proteases and Tulane virus protease. Data for HuNoV proteases sourced from reference 16. Data are presented as mean ± SEM of three independent experiments.

Previous studies have shown that TV-Pro cleaves the GI.1 substrates in the polyprotein, albeit with lower cleavage efficiency than that of GI.1-Pro (14). The same studies also demonstrated that GI.1-Pro cleaved the TV polyprotein substrate sequence, albeit with lower cleavage efficiency than that of the native protease. Using fluorogenic peptide substrates that represent the p48–p41 cleavage sequences of GI and GII HuNoVs, we assessed TV-Pro enzymatic activity against GI and GII HuNoV substrates. However, in our experiments, we found that the TV-Pro was more efficient at cleaving GI substrates than the GI.1-Pro and was within 20% of the cleavage efficiency of HuNoV GII proteases (Fig. 6).

Analysis of the TV-Pro kinetics against the GI substrate shows that the turnover rate and Michaelis constant are close to those of GI.1-Pro. However, the same analysis for TV-Pro against the GII substrate reveals a very low turnover rate (5-fold slower than the kcat of the GII.3 protease, the lowest kcat among GII proteases) and a low Michaelis constant (13.7 µM), possibly indicating that TV-Pro may have a product release bottleneck with this substrate (Fig. 6). Since the substrate changes occur at the P3–P4 position (FHLQ to YELQ), the difference in activity may stem from an additional gap in the S4 substrate pocket; however, further experiments are required to confirm any potential structural interactions involving a P4 tyrosine side chain. It is also possible that the P3 residue difference (His to Glu) may facilitate additional interactions with nearby residues such as Ser156 and Tyr157.

Given the solved structure of the complex between TV-Pro and rupintrivir, we expected rupintrivir to inhibit TV-Pro. We assayed the inhibition of TV-Pro by rupintrivir using the same continuous-time enzymatic assay in the presence of the norovirus GI substrate peptide, as described previously (16) and estimated rupintrivir’s covalent inhibition efficiency of TV-Pro to be 169.3 M^-1^s^-1^ (Fig. 6D). This is comparable to rupintrivir’s inhibition efficiency against GI.1-Pro and much higher than rupintrivir’s inhibition efficiency against GII proteases (293.9 M^-1^s^-1^ for GI.1 and 3.7 M^-1^s^-1^ for GII.4 Sydney, respectively (16).

### Rupintrivir inhibits TV replication in cultured cells

**Fig 7:**
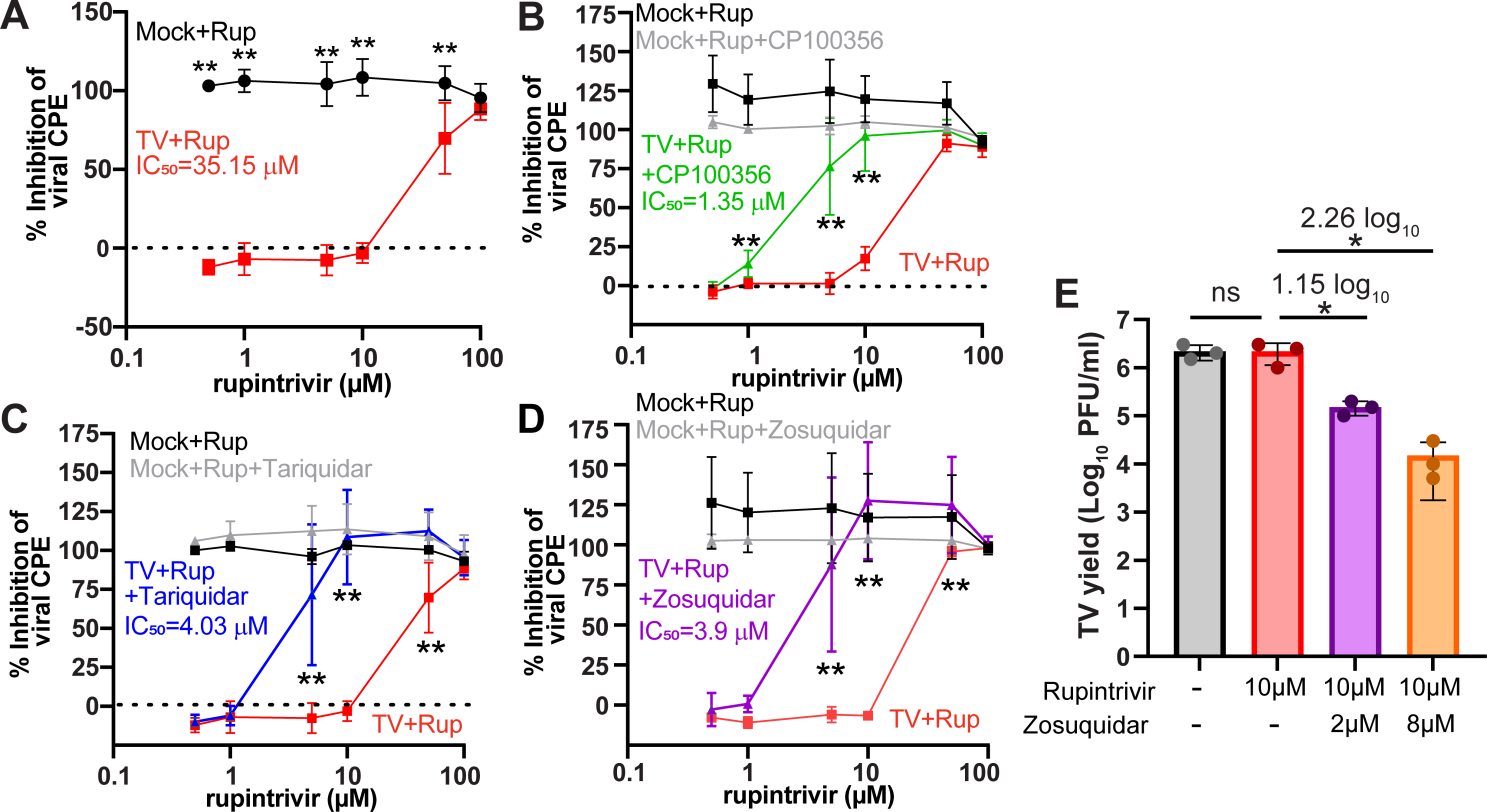
Antiviral effect of rupintrivir against Tulane virus is enhanced by P-glycoprotein inhibitors. (**A**) Percentage inhibition of monolayer clearance based on OD from eluted crystal violet of stained monolayers. Various concentrations of rupintrivir were used to treat mock- (black) and TV-infected (red) monolayers to determine the IC50 of rupintrivir. (**B–D**) Graphs depicting percentage inhibition of monolayer clearance comparing mock- and TV-infected monolayers treated either with various concentrations of rupintrivir alone (mock: black; TV: red) or in combination with the indicated P-glycoprotein inhibitor (mock: gray; TV: green, CP100356; blue, tariquidar; purple, zosuquidar). Graphs were generated in GraphPad Prism. The IC50 values shown were calculated by using nonlinear regression analysis. (**E**) TV yield from MA104 cells was measured without drug treatment, in the presence of 10 μM rupintrivir alone or with 10 μM rupintrivir plus zosuquidar. The experiments were conducted in biological triplicate, and values were plotted as mean ± SD. Statistical significance was calculated using unpaired *t*-test for panels **A–D** (**: *P* < 0.01) and using one-way ANOVA for panel E (*: *P* < 0.05).

Since TV has a robust cell culture replication system, we next examined whether rupintrivir inhibited TV replication and, if so, whether the inhibitory concentration and efficiency were similar to that of the *in vitro* assays. TV infection of MA104 cells is lytic and destroys the cell monolayer, so we used crystal violet staining of the monolayer after infection to determine the extent to which rupintrivir protected monolayer integrity as a measure of its antiviral activity against TV replication. First, we tested rupintrivir at concentrations ranging from 0.3 to 100 μM and found that rupintrivir exhibited modest antiviral activity against TV at concentrations above 10 μM, with a 50% inhibitory concentration (IC50) of ~35.15 μM (Fig. 7A). The modest activity of rupintrivir against TV in cell culture was somewhat surprising, given the *in vitro* studies (which show rapid inhibition within an hour even at 10 μM and below) and prior studies showing rupintrivir inhibition of murine norovirus replication in cell culture (7). One possible reason for this discrepancy is that rupintrivir is a substrate for multidrug efflux pumps, such as P-glycoprotein, as observed for other protease inhibitors (14). If so, adding a P-glycoprotein inhibitor would be expected to stabilize the cytosolic concentration of rupintrivir and increase its observed antiviral efficiency against TV. To test this, we repeated the rupintrivir inhibition assays in the absence or presence of three P-glycoprotein inhibitors: CP100356, tariquidar, and zosuquidar (Fig. 7B–D). We found that inclusion of these compounds significantly increased the antiviral effectiveness of rupintrivir against TV, decreasing the IC50 to 1.35 μM for CP100356, 4.03 μM for tariquidar, and 3.9 μM for zosuquidar. Importantly, neither rupintrivir nor any of the P-glycoprotein inhibitors exhibited cytotoxicity at the concentrations used. Finally, we assessed the extent to which rupintrivir reduced infectious virus yield. We found that while 10 μM rupintrivir alone had no effect, in the presence of 2 μM or 8 μM zosuquidar, TV yield was reduced by 1.15 log10 and 2.26 log10, respectively (Fig. 7E).

### Structural comparison with other recovirus proteases

**Fig 8:**
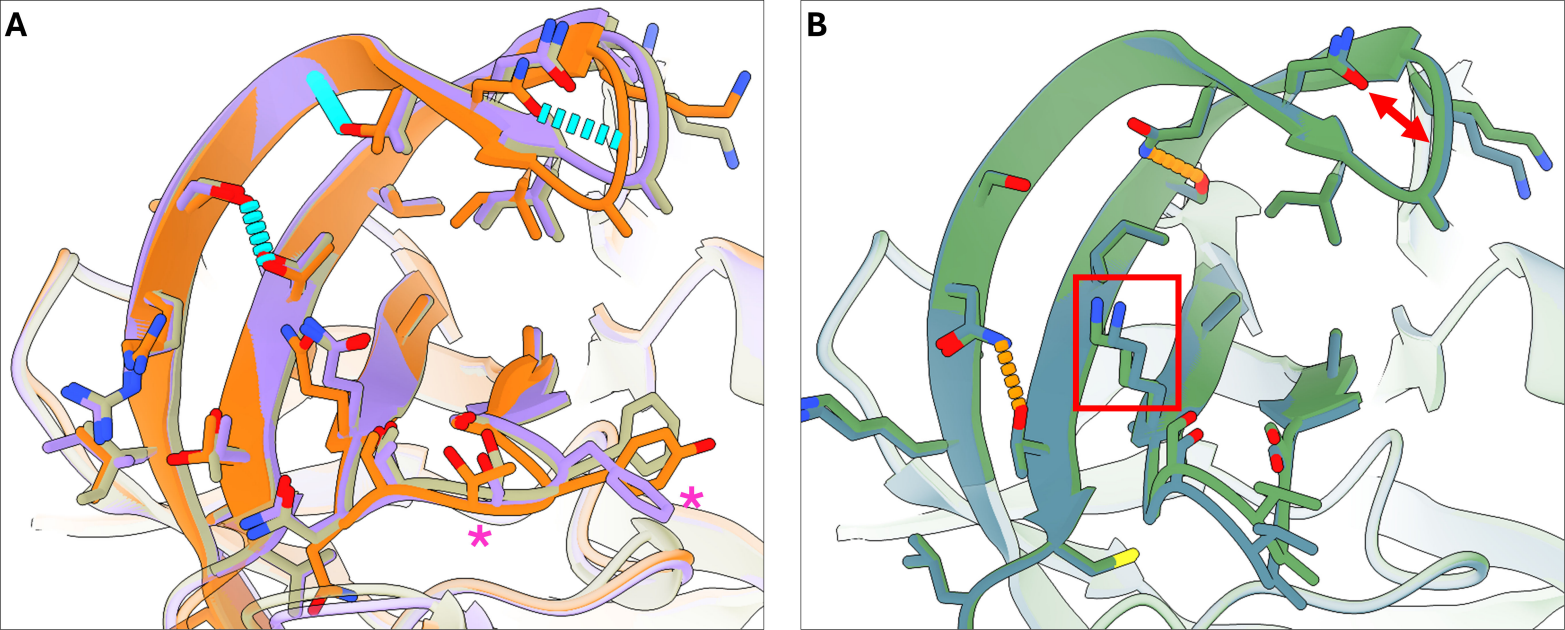
Comparison of the TV-Pro crystal structure with AlphaFold3-predicted recovirus protease structures. (**A**) The crystal structure of TV-Pro (orange) exhibits high similarity to the AlphaFold3-predicted structures of Mo-TG30 (purple) and WUHARV Calicivirus 1 (beige) proteases, with identical makeup of the S4 pocket with similar hydrogen bonding networks (cyan). Changes in the peripheral loops are marked with asterisks. (**B**) AlphaFold3-predicted structures of Bangladesh 2007 (blue) and Recovirus sp. (green) proteases are highly similar to each other but have a different hydrogen bonding network (hydrogen bonds in orange) supporting the BII-CII loop compared to TV-Pro (including one putative hydrogen bond location marked by the red arrow). However, the cleft in the S4 pocket appears to be preserved by the glutamine-to-lysine mutation, as marked by the red box.

To determine whether the structural features observed in the TV-Pro are present in other recovirus strains, we collected protease sequences from several recovirus strains and predicted their structures using AlphaFold 3 (19). TV was putatively classified as G1.1 recovirus, while WUHARV Calicivirus 1 and Recovirus Mo/TG30 strains were classified as G1.2 recovirus (20). The G1.2 recovirus proteases have 99% sequence identity with each other and 88% sequence identity with TV-Pro. The proteases from G3.1 recoviruses, Recovirus Bangladesh (2007) and Recovirus sp., share 94% sequence identity with each other and around 50% with the G1 recovirus strains. Despite the difference, for all strains, the predicted structures show high backbone homology of both beta barrels to TV-Pro. The G1 recovirus substrate-binding surfaces are virtually identical, with both the cleft and the three hydrogen bonds in the BII-CII loop preserved (Fig. 8A). G1.2 recovirus proteases differ from G1.1 TV-Pro at two residues peripheral to the S3 pocket: Y157F and T158S. The G3.1 recovirus proteases also show a preserved S4 pocket cleft, despite the Q161K mutation. However, the hydrogen bonding of the BII-CII loop changed substantially: The R98N mutation introduced a new hydrogen bond at the base of the BII-CII loop, the T114I mutation abolished a hydrogen bond, and the T103N/V112S mutation pair replaced a hydrogen bond (Fig. 8B). The loop residues peripheral to the S3 pocket also see substantial change (residues 157–160: YTTN to AVSG).

Out of the strains examined, Mo/TG30 may be of interest because one report showed the ability to replicate in human HEK293T cells, albeit to a lesser degree than simian cell lines (20). Given the minor differences between the substrate-binding sites of TV-Pro and Mo/TG30 recovirus protease, we expect the findings from this study for TV-Pro to apply to Mo/TG30 recovirus protease as well.

## DISCUSSION

### Feasibility of TV as a surrogate for HuNoV-Pro inhibitor screening

In this study, one of our goals was to determine whether TV and other recoviruses are suitable surrogates for cellular screening of HuNoV-Pro inhibitors. Traditionally, the effectiveness of HuNoV protease inhibitors has been tested in a cellular environment using norovirus replicons as a model (6–8). The replicons require protease activity for replication and retention within the cell, but this system does not comprehensively capture an infection environment. In the development of antivirals, a cellular model of viral infection ideally should provide insights into both a compound’s efficacy in an infection environment and its cellular permeability and pharmacokinetics. More recent advancements, such as the zebrafish larvae system and, especially, the human intestinal enteroid system, have provided more biologically relevant norovirus infection models for the screening of norovirus antivirals. However, both systems are unable to cultivate sufficiently high yields of infectious viral particles to passage the viral stock continually. Recently, a new breakthrough in the human intestinal enteroid system has enabled serial passaging of GII.3 norovirus and enhanced the replication of several other strains of norovirus, which increases the feasibility of using HIEs for antiviral drug screening (12). While the cost and complexity of HIEs and HIOs are potential barriers to their routine use in compound screening, recent developments have allowed these systems to be used in 96-well plates for drug testing, including in high-throughput screens (21–23), with the possibility to further scale the testing to 384-well plates.

While HuNoV models for drug screening are a “gold standard,” evaluation of surrogate viruses (e.g., Tulane virus) with traditional cell culture systems for the ability to identify antiviral compounds remains a potentially valuable tool. Here, we confirm previous reports (14) that TV-Pro can cleave the GI.1 HuNoV substrate and additionally demonstrate that TV-Pro can cleave the GII HuNoV substrate. While TV-Pro exhibits similar kinetics against the GI HuNoV substrate compared to GI.1-Pro, it shows high specificity, low-turnover cleavage kinetics of the GII HuNoV substrate compared to GII proteases. We also determined the crystal structures of TV-Pro, which provided valuable insights into substrate- and inhibitor-binding interactions. The S2 pocket and the new cleft identified in the S4 pocket of TV-Pro differ from those of HuNoV proteases and are likely correlated with differences in their respective substrate P2 and P4 side chain conformations. The cleft in the S4 pocket, in particular, is likely to lead to TV-Pro-bound inhibitor conformations that are dissimilar from HuNoV protease-bound inhibitor conformations. As a result, potential compounds may show differential activity against TV-Pro compared to the HuNoV protease. This may be further exacerbated in GII proteases as TV-Pro potentially has a more rigid BII-CII loop, resembling that of HuNoV GI proteases, compared to the flexible BII-CII loop in GII proteases. However, while the TV culture system and recovirus culture systems in general are not fully compatible surrogates for HuNoV proteases in their current state, they are still valuable tools to study the efficacy of protease inhibitors, especially when the effectiveness of the compounds can be evaluated in the context of the possible differences in their *in vitro* inhibition potencies against TV and HuNoV proteases.

Furthermore, the TV culture system is useful for testing combinations of protease inhibitors and other drugs, such as P-glycoprotein inhibitors. In our studies, we found that rupintrivir effectively inhibited TV protease *in vitro* and inhibited TV in cell culture, producing a 1–2 log10 reduction in virus yield, but the latter required the addition of a P-glycoprotein inhibitor to maximize the antiviral activity in the MA104 cell line model of TV infection. While P-glycoprotein is expressed in gastrointestinal epithelial cells, the overall effect on rupintrivir’s antiviral activity may vary based on P-glycoprotein expression levels. Previous studies have shown that 50 µM rupintrivir inhibited GII.4 HuNoV replication by ~1 log10 in fetal enteroids in the absence of any P-glycoprotein inhibitors (24). Our results with TV infection of MA104 cells similarly showed that 50 µM rupintrivir alone inhibited virus-induced cytopathic effect (CPE) by ~70%; however, the rupintrivir antiviral activity was significantly boosted by the addition of P-gp inhibitors, such that even lower rupintrivir doses (3–10 µM) exhibited moderate to strong antiviral activity. This underscores that rupintrivir could be a lead compound for optimization, and further studies will be required to assess the impact of P-glycoprotein-mediated drug efflux on rupintrivir derivatives for boosting the antiviral activity against human norovirus replication in more complex systems, including both adult and pediatric human intestinal enteroids.

### Potential modifications to enable the use of TV as a surrogate for protease inhibitor screening

Given that recombinant TV can be generated with reverse genetics (25, 26), the protease sequence of TV or other G1.1 recoviruses can be modified to generate surrogate viruses encoding proteases that exhibit specificity and substrate interactions similar to those of GI and GII proteases. For example, the cleft in the S4 pocket is generated by the gap between the side chains of Gln-161 and Ala-163. Mutating these to Thr-161 and Val-163 to match the amino acids at the same positions in HuNoV-Pro would eliminate this gap and potentially force the P4 side chain of TV-Pro substrates and inhibitors to bind in a conformation similar to those observed for HuNoV-Pro substrates and inhibitors. Additionally, to allow the BII–CII loop of TV-Pro to become more flexible, mutations that eliminate the hydrogen bonds between Ser-99/Thr-114, Thr-103/Gly-101, and Asn-104/Gly-107 can also be introduced.

Since HuNoV proteases have been shown to cleave TV-Pro substrates (14), it may be possible to generate chimeric recoviruses containing the HuNoV protease sequence in place of the TV-Pro sequence in the viral genome. The heterologous HuNoV protease would still cleave the TV polyprotein substrate, albeit at a reduced rate. The substrate cleavage sites may be altered along with the protease sequence, or they may also be adapted upon repeated passages of the recombinant virus.

In summary, our studies show that the TV culture systems demonstrate significant utility in screening HuNoV-Pro inhibitors and their combinations with other drugs. While the structural differences between TV-Pro and HuNoV-Pro mean that TV-Pro is not a fully compatible surrogate for the latter, the structural and biochemical data presented here enabled us to identify potential strategies to modify the TV-Pro using reverse genetics, thereby creating more effective surrogate viruses for screening HuNoV-Pro inhibitors in the future.

## MATERIALS AND METHODS

### Expression and purification of TV-Pro

The Tulane virus protease (YP_009666335.1) sequence was synthesized by Genscript and subcloned into a pET vector downstream of a His-TELSAM-HRV 3C cut site sequence, as described previously (16, 27). For the TV-Pro structure with no exogenous ligands, as well as the enzymatic assays, an additional tryptophan was added to the C-terminus of the protease sequence for concentration measurement with UV absorbance. The plasmid was transformed into XJb (DE3) *E. coli* (Zymo Research) and grown overnight at 30°C in Terrific Broth (TB) supplemented with 50 µg/mL kanamycin and 1.5% glucose. The overnight culture was used to inoculate TB grown at 37°C, supplemented with kanamycin, 0.4% glycerol, 2 mM MgCl2, and 0.05% glucose, and protein expression was induced by adding IPTG to 0.4 mM when the optical density reached 0.7. The culture was grown at 18°C for 16 h before harvesting by centrifugation at 3,500 × *g* for 30 min. The cell pellet was resuspended in lysis buffer (20 mM HEPES, 500 mM NaCl, and 1 mM TCEP, pH 7.5) supplemented with GENIUS Nuclease (ACROBiosystems) and lysed with the LM-20 microfluidizer (Microfluidics) at a pressure of 18,000 PSI. The lysate was clarified by centrifugation at 35,000 × *g* for 30 min. Ni-NTA resin was equilibrated in lysis buffer and incubated with the clarified lysate and left at 4°C with rocking for 1 h. The resin was then washed four times with wash buffer (20 mM HEPES, 1 M NaCl, 30 mM imidazole, and 1 mM TCEP, pH 7.5) before transferring to a glass Econo-Column (Bio-Rad). The column was washed once with lysis buffer before 6 column volumes of elution buffer (20 mM HEPES, 150 mM NaCl, 300 mM imidazole, and 1 mM TCEP, pH 7.5) were applied to elute the protein. The eluted protein was then mixed with 3C protease (pET-NT*-HRV3CP was a gift from Gottfried Otting; Addgene plasmid #162795; https://www.addgene.org/162795/; RRID:Addgene_162795) at a 50:1 wt/wt ratio, before the mixture was transferred to SnakeSkin dialysis tubing (7K MWCO, ThermoFisher) and dialyzed overnight (16 h) with stirring in dialysis buffer (10 mM HEPES, 100 mM NaCl, 15 mM imidazole, and 1 mM TCEP, pH 8.0). The mixture was applied to Ni-NTA resin pre-equilibrated with size exclusion chromatography (SEC) buffer (10 mM HEPES, 50 mM NaCl, 1 mM TCEP, pH 8.0) and incubated for 80 min at 4°C with rocking. The flow-through was collected and concentrated with the Amicon Ultra-15 (10K MWCO, MilliporeSigma) before injection on a HiLoad 16/60 Superdex 75 pg column equilibrated in SEC buffer. The pooled fractions containing the protease were concentrated and used immediately, while the remaining samples were supplemented with 50% glycerol to a final glycerol concentration of 10%, then concentrated to around 10 mg/mL when possible, and subsequently flash-frozen in liquid nitrogen and stored at −80°C.

### Crystallization, data collection, and refinement

Forty microliters of 40 mM rupintrivir (MilliporeSigma) were diluted in 14 mL of SEC buffer, before 500 µL of TV-Pro (without tryptophan) at 5.5 mg/mL concentration (as measured by Bradford assay) was added. The sample was left to incubate overnight with gentle rocking at 4°C, concentrated until the UV280 absorbance of the sample was around 2.5, and used to set up crystallization by sitting drop plates (96-well Intelli-plate crystallization plates, Art Robbins) using the Mosquito liquid handler (SPT Labtech). Each drop contains 0.2 µL protein and 0.2 µL of crystallization solution. The plate was then sealed with Crystal Clear Sealing Film (Hampton Research). For TV-Pro crystals without added ligands, TV-Pro (with one added tryptophan in sequence) was concentrated to 12 mg/mL (calculated from UV280 absorbance) and used to set up 0.4 µL sitting drops, as described above.

TV-Pro-rupintrivir crystals formed 2 weeks after setting up, while a crystal of TV-Pro without ligands formed after a month. The crystallization conditions are listed in Table S1. The crystals were harvested and immediately flash-frozen without additional cryoprotection and sent to synchrotron beamline facilities at the Advanced Light Source (Beamline 8.2.1, TV-Pro with rupintrivir; Beamline 8.2.2, TV-Pro without added ligands) for data collection.

Crystal diffraction images were processed with the *xia2* package (28) using either the dials (29, 30) or XDS (31) pipeline through CCP4 (32, 33). For the Tulane virus protease-rupintrivir structure, a predicted structure was generated using the AlphaFold2 pipeline (34–36) built into Phenix (37) and subsequently used for molecular replacement with PHASER (38). Refinement with phenix.refine and ligand restraints (ReadySet) was done in Phenix (37, 39–42), with manual model building done in COOT (43). For TV-Pro without an added ligand structure, the TV-Pro-rupintrivir model was used for molecular replacement in PHASER and refined with Phenix and Coot. The resolution cutoff was determined by using mean I/sigmaI ~ 2, as calculated by phenix.merging_statistics (37), as a cutoff. Data collection and refinement statistics are listed in Table S2. Protein structures were prepared in ChimeraX (44–46).

### Enzymatic and time-dependent inhibition assay

The activity of TV-Pro and its inhibition by rupintrivir are measured using the fluorescent resonance transfer assay described previously (16, 17, 47). Fluorogenic substrate peptides corresponding to the cleavage site between p48 and p41 subunits in the viral polyprotein [GI.1: Glu(EDANS)-PDFHLQGPEDLA-Lys(Dabcyl), GII: Glu(EDANS)-GDYELQGPEDLA-Lys (Dabcyl)] were synthesized by GenScript USA Inc. For enzymatic activity on the GI substrate, 1.2 mM GI substrate solution in dimethyl sulfoxide (DMSO) was diluted to 256 µM in assay buffer (10 mM HEPES, 30% glycerol, 0.1% CHAPS, and 10 mM DTT, pH 8.0) and serially diluted 2-fold down to 1 µM. A protease solution (4 µM stock solution for the GII substrate and 1.2 µM for the GI substrate) was prepared by diluting a stock protease solution in assay buffer. Fifty microliters of protease and substrate solution was dispensed into each well of 96-well black NBS plates (Corning 3991) and briefly mixed with a multichannel pipette before shaking for 90 s at 37°C at 1,000 RPM in the Flexstation 3 multimode plate reader (Molecular Devices). Assays were run for 2 h at 37°C, and the signal was measured every 90 s (excitation wavelength: 340 nm, emission wavelength: 490 nm, filter: 475 nm). The product concentration was converted from the measured relative fluorescence units using a standard curve. Initial velocities of each condition were calculated by linear regression in GraphPad Prism 8 (GraphPad Software Inc.). Nonlinear regression analysis in GraphPad Prism 8 was used to fit Michaelis constants (K_m_), catalytic constants (k_cat_), and catalytic efficiency (k_cat_/K_m_).

For the inhibition assay, a 100 µM GI substrate solution was prepared by diluting a 1.2 mM GI substrate stock in DMSO into assay buffer. A 1.2 µM stock solution of the protease was prepared by diluting the concentrated stock protease into assay buffer. A series of rupintrivir stock solutions (400, 300, 200, 150, 100, 75, and 50 µM, corresponding to 10× final concentrations) was prepared by serially diluting a 40 mM rupintrivir DMSO stock solution into assay buffer. Fifty microliters of substrate and 10 µL of the inhibitor were dispensed into each well of a black 96-well NBS plate (Corning 3991). Forty microliters of the stock protease solution was then added and briefly mixed with a multichannel pipette before the plate was sealed with ClearSeal film (Hampton Research) and shaken for 90 s at 1,000 RPM at 37°C. Assays were run for 4 h and measured every 90 s. The relative fluorescence units over time (F vs T) curves were fitted using the scipy.optimize (48) Python library using equation 1. Initial parameters for steady-state velocity (V_s_), initial velocity (V_0_), offset (F_0_), and observed covalent inhibition rate (k_obs_) were estimated with the differential_evolution function and then fitted with the curve_fit function using the bounded “trf” option.


(1) $$F=F_{0}+V_{s}t+\left(V_{0}-V_{s}\right)\times \frac{1-e^{-k_{obs}t}}{k_{obs}}$$

The obtained k_obs_ values were imported into GraphPad Prism 8 and used for fitting with nonlinear regression using equation 2 to obtain k_inact_ and K_I_^app^, with [I] as the inhibitor concentration and k_ctrl_ as the observed degradation rate in the control samples with only the protease and substrate. The correction for substrate competition was done using equation 3, with [S] as the substrate concentration and K_m_ as the Michaelis constant for the TV-Pro–GI substrate pair.


(2) $$k_{obs}=k_{ctrl}+\frac{k_{inact}\left[I\right]}{K_{I}^{app}+\left[I\right]}$$

(3) $$\frac{k_{inact}}{K_{I}}=\frac{k_{inact}}{K_{I}^{app}}\left(1+\frac{\left[S\right]}{K_{m}}\right)$$

### Structural prediction and comparison of recovirus proteases

In addition to the TV-Pro sequence, recovirus protease sequences were extracted from recovirus genomes, as compiled in reference 20, for Mo/TG30 (OQ184950.1), Recovirus sp. (MG571787.1), WUHARV Calicivirus 1 (JX627575.1), and Recovirus Bangladesh/289/2007 (JQ745645.1) (20, 49–51) by centering around the GDCG catalytic motif within nonstructural polyprotein sequences. Initial predictions were made using the AlphaFold Server (19), and subsequently, the sequences were truncated to only the globular protease domain. A final round of predictions was made using the AlphaFold Server, and the resulting predicted structures all have pTM scores of 0.88 or higher. The predicted structures are then aligned using the Matchmaker tool and visualized in ChimeraX (44–46, 52).

### Viral cytopathic effect inhibition assay

The CPE inhibition assay was performed as previously described (26). MA104 cells were grown to confluency in 24-well tissue culture plates. The confluent monolayers were infected with Tulane virus (MOI 10) for 1 h, followed by PBS wash, and then rupintrivir-containing media was added onto the monolayer at the indicated concentrations. Mock-infected wells with increasing concentration of rupintrivir were used as a negative control. DMSO-treated uninfected and infected wells were used to normalize the calculation of the percent inhibition of TV-induced cytopathic effect. For wells with P-glycoprotein inhibitor, 2 µM of the indicated P-glycoprotein inhibitor along with rupintrivir was added into media. Monolayers were incubated for 48 h, and then a PBS wash was performed followed by crystal violet staining for 10 min on a rocking shaker. Monolayers were washed to remove unbound stain and dried at room temperature. The bound crystal violet stain was dissolved by adding 500 µL of 33% acetic acid and kept on a rocker shaker for 10 min. Then, 100 µL of the dissolved stain was added into a 96-well plate, and the absorbance was measured at 630 nm (BioTek ELx808 spectrophotometer).

The percent inhibition of virus cytopathic effect was calculated with the following equation: inhibition rate = [(OD inhibitor-treated infected cells − OD DMSO-treated infected cells) / (OD DMSO-treated uninfected cells − OD DMSO-treated infected cells)] × 100.

### Tulane virus yield assay

For viral yield assay, confluent MA104 monolayers seeded in 24-well plate were infected with Tulane virus (MOI 10) for 1 h, and the media was replaced with media containing either 10 µM rupintrivir or rupintrivir + zosuquidar (concentrations as indicated). DMSO-containing infected wells were used as negative controls. At 24 h post-infection, the plates were harvested by two freeze/thaw cycles, and the supernatant was collected to determine the viral titer by plaque assay, as previously described (26).

## Data Availability

The data supporting the findings of this study are available within the article and its supplemental material (Fig. S1; Tables S1 and S2). All the data pertaining to crystallographic structures are deposited in the Protein Data Bank with the PDB IDs as noted in Table S2.

## SUPPLEMENTAL MATERIAL

Supplemental material jvi.02176-25-s0001.docx Tables S1 and S2; Fig. S1.

ASM does not own the copyrights to Supplemental Material that may be linked to, or accessed through, an article. The authors have granted ASM a non-exclusive, world-wide license to publish the Supplemental Material files. Please contact the corresponding author directly for reuse.
