# Supplementary material for: Tulane virus protease as a structural surrogate for inhibitor screening of human norovirus proteases
Source: J Virol. 2026 Feb 11;100(3):e02176-25. doi: 10.1128/jvi.02176-25 (PMC13011392; doi:10.1128/jvi.02176-25)
Supplement: Supplemental material — Tables S1 and S2; Fig. S1. [file jvi.02176-25-s0001.docx]

# Supplemental Materials for

# TULANE VIRUS PROTEASE AS A STRUCTURAL SURROGATE FOR INHIBITOR SCREENING OF HUMAN NOROVIRUS PROTEASES

**Son Pham^1^, Nikhil Sharma^2^, Banumathi Sankaran^3^, Jalen Nguyen^1^, Mary K. Estes^2,4^, Joseph M. Hyser^2,5^, B.V. Venkataram Prasad^1,2*^**

^1^Verna and Marrs McLean Department of Biochemistry and Molecular Pharmacology, Baylor College of Medicine, Houston, TX, USA

^2^Department of Molecular Virology and Microbiology, Baylor College of Medicine, Houston, TX, USA

^3^Berkeley Center for Structural Biology, Molecular Biophysics, and Integrated Bioimaging, Lawrence Berkeley Laboratory, Berkeley, CA, USA

^4^Department of Medicine, Baylor College of Medicine, Houston, TX, USA

^5^Alkek Center for Metagenomics & Microbiome Research, Baylor College of Medicine, Houston, Texas, USA

^*^Corresponding author: vprasad@bcm.edu

**This file includes:**

Tables S1 and S2

Figure S1

Table S1. Crystallization conditions

| Tulane virus protease without added ligands | 0.1M amino acids mix, 1.2% cholic acid derivative mix, 0.1M Buffer system 1 pH 6.5, 37.5% Precipitant Mix 4 (Morpheus Fusion screen condition E5, Molecular Dimensions) |
| --- | --- |
| Tulane virus protease with rupintrivir | 0.3M Ammonium citrate dibasic, 0.1M Magnesium formate, pH 6, 22.5% (v/v) PurePEGs Cocktail |

Table S2. Crystallographic data collection and refinement statistics

|  | **TulanePro_Substrate** | **TulanePro_Rupintrivir** |
| --- | --- | --- |
| PDB ID | 9ZFN | 9ZFQ |
| Wavelength (Å) | 1.0002 | 1.00013 |
| Resolution range (Å) | 45.67 – 2.1 (2.15 - 2.10) | 45.91 - 1.9 (1.94 - 1.9) |
| Space group | P 2_1_ 2_1_ 2_1_ | P 2 2_1_ 2_1_ |
| Unit cell (a, b, c, α, β, γ) | 56.28 Å, 78.16 Å, 86.8 Å,  90°, 90°, 90° | 38.792Å, 50.482Å, 91.813Å,  90°, 90°, 90° |
| Total reflections | 219883 (16811) | 153653 (8426) |
| Unique reflections | 19959 (1490) | 14834 (1002) |
| Multiplicity | 11.0 (11.3) | 10.4 (8.4) |
| Completeness (%) | 86.8 (99.1) | 100.0 (100) |
| Mean I/sigma(I) | 20.5 (3.6) | 15.2 (2.6) |
| Wilson B-factor (Å^2^) | 32.5 | 19.29 |
| R-merge | 0.081 (0.701) | 0.083 (0.232) |
| R-meas | 0.085 (0.734) | 0.087 (0.248) |
| R-pim | 0.025 (0.214) | 0.027 (0.084) |
| CC1/2 | 0.999 (0.889) | 0.998 (0.965) |
| CC* | 1 (0.970) | 1 (0.991) |
| Reflections used in refinement | 19948 (1490) | 14833 (1002) |
| Reflections used for R-free | 998 (74) | 905 (61) |
| R-work | 0.2038 (0.2444) | 0.1781 (0.2268) |
| R-free | 0.2351 (0.2755) | 0.2127 (0.2793) |
| CC (work) | 0.957 (0.865) | 0.962 (0.925) |
| CC (free) | 0.899 (0.783) | 0.942 (0.937) |
| Number of non-hydrogen atoms | 2687 | 1400 |
| macromolecules | 2553 | 1226 |
| ligands | 0 | 43 |
| solvent | 134 | 131 |
| Protein residues | 351 | 170 |
| RMS (bonds) | 0.005 | 0.007 |
| RMS (angles) | 0.72 | 1 |
| Ramachandran favored (%) | 97.41 | 96.43 |
| Ramachandran allowed (%) | 2.59 | 3.57 |
| Ramachandran outliers (%) | 0 | 0 |
| Rotamer outliers (%) | 0 | 0.73 |
| Clashscore | 3.27 | 1.94 |
| Average B-factor (Å^2^) | 36.52 | 24.5 |
| macromolecules | 36.38 | 23.55 |
| ligands |  | 24.67 |
| solvent | 39.22 | 33.36 |

**
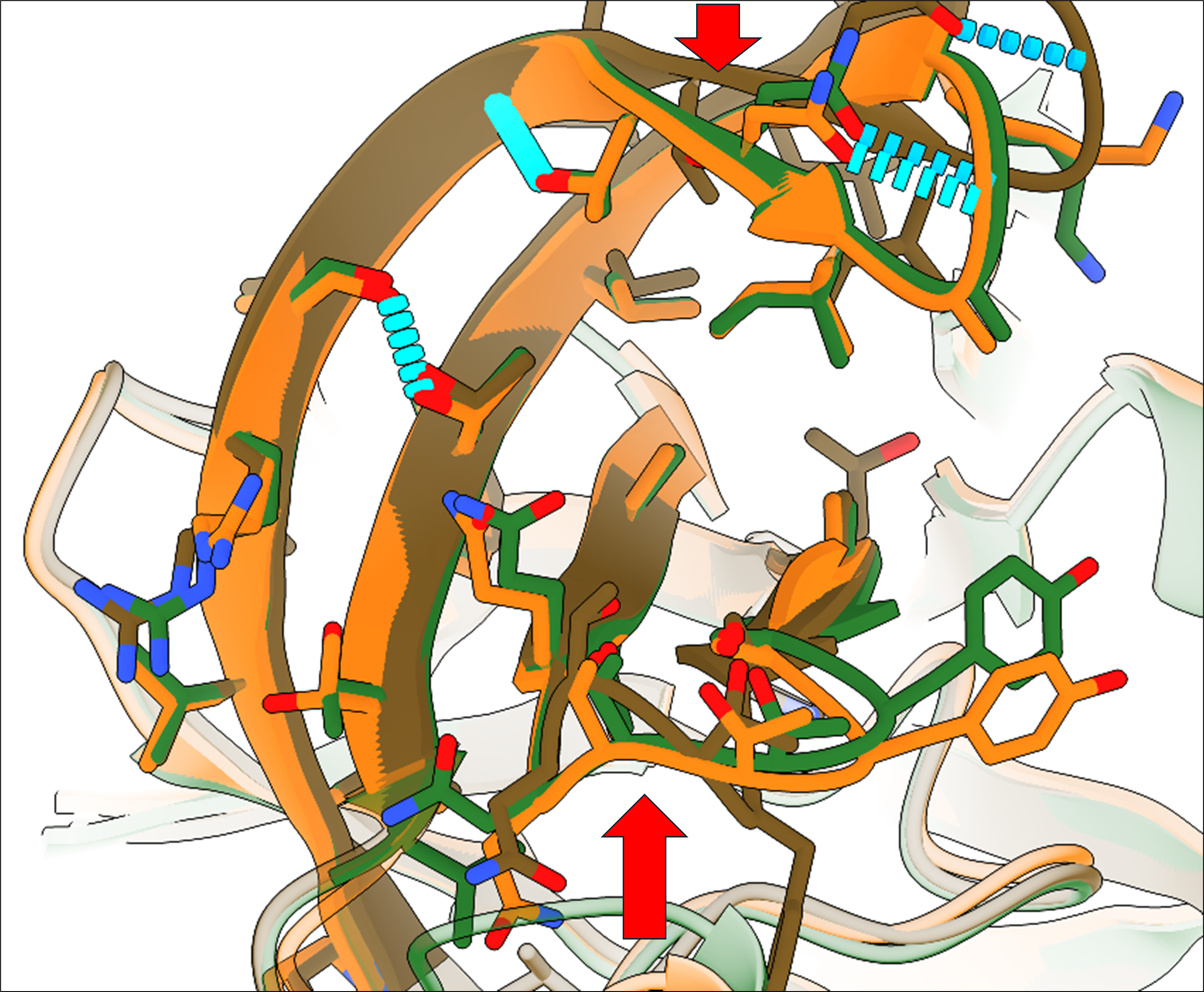
**

**Supplementary Figure S1. Comparison of crystal structure of Tulane virus protease without added ligands and AlphaFold3 and AlphaFold2 predicted structures of Tulane virus protease.**

AlphaFold3-predicted structure of Tulane virus protease (green) is well-aligned with the crystal structure of Tulane virus protease without added ligands (orange), including the three hydrogen bonds in the BII-CII loop (cyan), as well as the conformations of the BII-CII loop (top, red arrow) and the S4 pocket peripheral loop (bottom, red arrow). In contrast, AlphaFold2-predicted structure of Tulane virus protease (brown), which was used for phasing, predicted differential conformations for both the BII-CII loops and the peripheral loops, including a differential conformation for Thr103 (top, red arrow) that eliminates a hydrogen bond in the BII-CII loop.
